# DNA methylation profiling to predict recurrence risk in stage Ι lung adenocarcinoma: Development and validation of a nomogram to clinical management

**DOI:** 10.1111/jcmm.15393

**Published:** 2020-06-12

**Authors:** Xianxiong Ma, Jiancheng Cheng, Peng Zhao, Lei Li, Kaixiong Tao, Hengyu Chen

**Affiliations:** ^1^ Department of Gastrointestinal Surgery Union Hospital Tongji Medical College Huazhong University of Science and Technology Wuhan China; ^2^ Department of Cardiovascular Surgery Union Hospital Tongji Medical College Huazhong University of Science and Technology Wuhan China; ^3^ Department of Hepatobiliary surgery Union Hospital Tongji Medical College Huazhong University of Science and Technology Wuhan China; ^4^ Department of Breast and Thyroid Surgery Union Hospital Tongji Medical College Huazhong University of Science and Technology Wuhan China; ^5^ Department of Pancreatic Surgery Union Hospital Tongji Medical College Huazhong University of Science and Technology Wuhan China; ^6^ NHC Key Laboratory of Hormones and Development Tianjin Institute of Endocrinology Tianjin Medical University Chu Hsien‐I Memorial Hospital Tianjin China

**Keywords:** DNA methylation, LUAD, nomogram, recurrence‐free survival, signature

## Abstract

Increasing evidence suggested DNA methylation may serve as potential prognostic biomarkers; however, few related DNA methylation signatures have been established for prediction of lung cancer prognosis. We aimed at developing DNA methylation signature to improve prognosis prediction of stage I lung adenocarcinoma (LUAD). A total of 268 stage I LUAD patients from the Cancer Genome Atlas (TCGA) database were included. These patients were separated into training and internal validation datasets. GSE39279 was used as an external validation set. A 13‐DNA methylation signature was identified to be crucially relevant to the relapse‐free survival (RFS) of patients with stage I LUAD by the univariate Cox proportional hazard analysis and the least absolute shrinkage and selection operator (LASSO) Cox regression analysis and multivariate Cox proportional hazard analysis in the training dataset. The Kaplan‐Meier analysis indicated that the 13‐DNA methylation signature could significantly distinguish the high‐ and low‐risk patients in entire TCGA dataset, internal validation and external validation datasets. The receiver operating characteristic (ROC) analysis further verified that the 13‐DNA methylation signature had a better value to predict the RFS of stage I LUAD patients in internal validation, external validation and entire TCGA datasets. In addition, a nomogram combining methylomic risk scores with other clinicopathological factors was performed and the result suggested the good predictive value of the nomogram. In conclusion, we successfully built a DNA methylation‐associated nomogram, enabling prediction of the RFS of patients with stage I LUAD.

## 1 INTRODUCTION

Lung cancer is one of the cancers with the leading cause of cancer‐related death worldwide.^1^ The majority of lung cancer is non‐small cell lung cancer (NSCLC). NSCLC is divided into three main subtypes including lung adenocarcinoma (LUAD), lung squamous cell carcinoma (LSCC) and large‐cell carcinoma.^2^ At present, LUAD is the most common histological subtype of lung cancer.^3^

The prognosis of patients with lung cancer is significantly associated with different TNM clinical stages. Early‐stage (IA‑ⅡB) NSCLC accounts for only 25%‐30% of all lung cancers.^4^ Surgery remains the primary treatment for operable and resectable stage I LUAD. However, about 20% of patients with stage I LUAD develop cancer recurrence after surgery.^5^ Therefore, an effort to identify effective biomarkers for prognosis of stage I LUAD is urgently required.

It has been revealed that genes controlled by DNA methylation were relevant to tumour development.^6^, ^7^ Numerous researches reported that DNA methylation may serve as potential prognostic biomarkers. For example, Guo et al reported that a five‐DNA methylation signature served as a novel prognostic biomarker in patients with ovarian serous cystadenocarcinoma.^8^ Sailer et al suggested that intragenic DNA methylation of PITX1 and the adjacent long non‐coding RNA C5orf66‐AS1 functioned as prognostic biomarkers in patients with head and neck squamous cell carcinomas.^9^ Sailer et al revealed that PITX2 DNA methylation may serve as a prognostic biomarker in patients with head and neck squamous cell carcinoma.^10^ Uhl et al indicated that DNA methylation of PITX2 and PANCR served as prognostic for overall survival in patients with resected adenocarcinomas of the biliary tract.^11^ DNA methylation was relevant to carcinogenesis by inhibiting the expression of the tumour suppressor gene and enhancing the expression of oncogenes.^12^, ^13^, ^14^, ^15^ Thus, the cancer tissues have a more remarkable DNA methylation pattern than that in normal tissues. In addition, DNA methylation patterns belong to inherently reversible changes and thus may be potential targets for drug therapy.^16^ Therefore, investigations on DNA methylation are promising in identifying predictive biomarkers for treatments and may help offer individualized treatments and prolong patients' survival time.

However, the utility of genome‐wide methylation analysis in clinical practice is restricted by the large sets of DNA methylation determined and the difficulties in complicated statistical analyses. In addition, the stability of prognostic methylation marker identified is restricted by different samples and the lack of regulation for primary confounding factors.^17^ Therefore, the whole‐genome methylation profiles of tumour tissues from patients with stage I LUAD were obtained from TCGA database and GEO database and a predictive risk model for RFS according to methylation of DNAs was established and examined via a bioinformatics approach in this study.

## 2 MATERIALS AND METHODS

### 2.1 DNA methylation data of stage I LUAD patients

All TCGA stage I LUAD DNA methylation data analysed by Illumina Human Methylation 450 BeadChip (Illumina Inc, San Diego, CA, USA) and clinical data were retrieved by using R TCGAbiolinks package.^18^ GSE39279 dataset and corresponding clinical information were obtained by using GEOquery package.^19^ DNA methylation levels were expressed as *β*‐values, calculated as *M*/(*M* + *U* + 100), in which *M* represented for the signal from methylated beads, and *U* represented the signal from unmethylated beads at the targeted CpG site. The methylomic data that matched with patient samples containing complete clinical recurrence survival information were selected to assess the association between DNA methylation levels and the associated RFS in stage I LUAD. Overall, 268 samples with 485 577 DNA methylation sites were analysed in our study. These 268 samples were divided into two cohorts: the first two‐thirds 70% served as the training cohort for identifying and establishing prognostic biomarkers, and the remaining 30% served as an internal validation cohort for confirming the predictive ability of the biomarker. In addition, the 118 stage I LUAD samples from GEO database (GSE39279) were analysed as an external validation cohort. LASSO method was used for identifying the significant methylation sites to predict prognosis of stage I LUAD patients. At the same time, LASSO Cox regression model was conducted via a publicly available R package ‘glmnet’^20^ for 1000 iterations.

### 2.2 Data processing, normalization and identification of differentially expressed methylation sites

Pre‐processing the data before constructing the prediction model was essential. Methylation sites whose beta value was not available (NA) in any specimens were excluded from our study. Then, we normalized the data with ‘betaqn’ function from wateRmelon package.^21^

Furthermore, all the patient specimens were separated into recurrent group and no recurrent group based on recurrence status. The normalized beta was transformed to *M* value on the basis of the formulation: *M* = log(*β*/(1 − *β*)). *M* value was applied to eliminate the bias caused by various probes. Then, *M* value was used to determine differentially expressed methylation sites between recurrence group and no recurrence cohorts with ‘dmpFinder’ function from minfi package.^22^

### 2.3 Statistical analyses

Relapse‐free survival was defined as the time from the beginning of treatment to the earliest local recurrence, distant metastasis and death. The univariate Cox proportional hazard analysis was acted in the training dataset to determine methylation sites significantly (*P* < .01) relevant to patient RFS as potential indicators. Then, the potential indicators were used to perform the LASSO Cox regression analysis for further identifying the candidate factors influencing the RFS of patients. Subsequently, the identified candidate markers were used as covariates to establish multivariate Cox proportional hazard model. Eventually, a 13‐DNA methylation signature was identified for predicting prognosis of stage I LUAD. Then, AUC was applied to weigh the model performance with the ‘survivalROC’ package. A formula was constructed to measure RFS risk scores for every patient on the basis of the model. Patients with stage I LUAD were separated into high‐ and low‐risk group with the median score as the cut‐off. Kaplan‐Meier survival analysis was executed to weigh the differences in RFS between the two cohorts, and Kaplan‐Meier curves were drawn via the ‘survival’ package.^23^

### 2.4 Construction of the nomogram

To improve the quality with a quantitative tool, we developed a nomogram on the basis of the ‘rms’ R package.^24^ The univariate Cox proportional hazard analysis and multivariate Cox proportional hazard analysis were performed based on methylation risk score and other clinicopathological factors. The factors with *P* ≤ 0.05 from multivariate Cox proportional hazard analysis were used to construct nomogram. Hazard ratios (HR) and corresponding 95% confidence interval (CI) were evaluated according to Cox proportional hazard models. The prognostic ability of the nomogram was weighed by C‐index, ROC and calibration plots.

## 3 RESULTS

### 3.1 Clinical characteristics of the study populations

**FIGURE 1:**
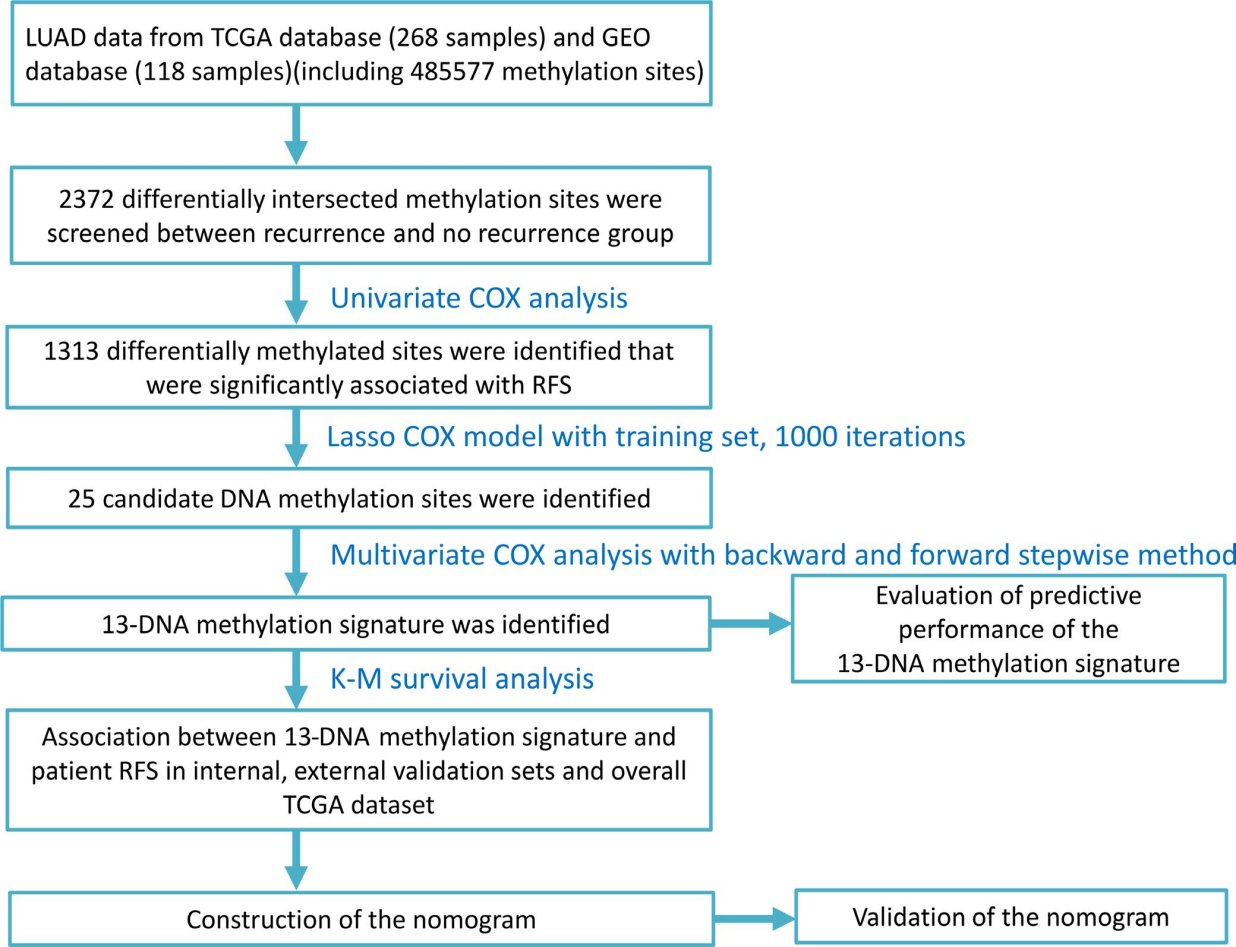
Flowchart of the present study

The study was performed on 268 TCGA patients and 118 GEO patients who were clinically and pathologically diagnosed with stage I LUAD. Of these TCGA patients, 111(41.42%) were male and 157(58.58%) were female. The median age at diagnosis was 66 years (range, 33‐88), respectively, and the median RFS was 595.5 days. The 3‐year RFS rate of all patients was 22.4%. The pathologic stage was defined based on the American Joint Committee on Cancer (AJCC) Cancer staging manual. The stage of stage I LUAD patients included stage I, stage IA, stage IB and 5(1.87%) patients in state I (stage I: whether stage IA or stage IB was not identified), 133(49.63%) patients in stage IA and 130(48.51%) patients in stage IB. Patients were divided into three groups based on location of tumour, including central lung 27(10.07%), peripheral lung 50(18.66%) and Not Available 191(71.27%), respectively. Anatomic neoplasm subdivision included L‐Lower, L‐Upper, R‐Lower, R‐Middle, R‐Upper and Not Available. R‐Upper group was the most common type 113(42.16%). Furthermore, race list group included American‐Indian or Alaska native, Asian, Black or African‐American, White and not available. White group was the most common type 215(80.22%). In addition, smoking history of stage I LUAD patients included smoking group, no smoking group and not available group. Smoking group was the most common type 173(64.6%). The demographic characteristics of stage I LUAD patients in TCGA dataset as well as GEO dataset were summarized in Table 1, and the overall design and flowchart of this study were displayed in Figure 1.

**TABLE 1:** Clinical characteristics of included patients

Characteristics	Total	Training dataset (n = 188)	Testing dataset (n = 80)	External validation test (n = 118)
Sex				
Female	157 (58.58)	107 (56.91)	50 (62.5)	61 (51.7)
Male	111 (41.42)	81 (43.09)	30 (37.5)	57 (48.3)
Age				
≤65	130 (48.51)	89 (47.34)	41 (51.25)	58 (49.2)
>65	131 (48.88)	94 (50)	37 (46.25)	60 (50.8)
Not available	7 (2.61)	5 (2.66)	2 (2.5)	
Smoking history				
Yes	173 (64.6)	122 (64.9)	51 (63.75)	99 (83.9)
No	90 (33.6)	63 (33.5)	27 (33.75)	16 (13.6)
Not available	5 (1.8)	3 (1.6)	2 (2.5)	3 (2.5)
Stage				
Stage I	5 (1.87)	4 (2.13)	1 (1.25)	10 (8.5)
Stage IA	133 (49.63)	86 (45.74)	47 (58.75)	99 (83.9)
Stage IB	130 (48.51)	98 (52.13)	32 (40)	9 (7.6)
Tumour				
T1	136 (50.75)	88 (46.81)	48 (60.00)	75 (63.6)
T2	132 (49.25)	100 (53.19)	32 (40.00)	43 (36.4)
Location in lung parenchyma				
Central lung	27 (10.07)	15 (7.98)	12 (15)	
Peripheral lung	50 (18.66)	34 (18.09)	16 (20)	
Not available	191 (71.27)	139 (73.94)	52 (65)	
History of neoadjuvant treatment				
No	268 (100)	188 (100)	80 (100)	
Anatomic neoplasm subdivision				
L‐lower	39 (14.55)	29 (15.43)	10 (12.5)	
L‐upper	64 (23.88)	40 (21.28)	24 (30)	
R‐lower	39 (14.55)	24 (12.77)	15 (18.75)	
R‐middle	8 (2.99)	4 (2.13)	4 (5)	
R‐upper	113 (42.16)	89 (47.34)	24 (30)	
Not available	5 (1.87)	2 (1.06)	3 (3.75)	
Residual_tumour				
R0	172 (64.18)	118 (62.77)	54 (67.5)	
R1	1 (0.37)	1 (0.53)		
RX	95 (35.44)	69 (36.71)	26 (32.5)	
Race				
Asian	4 (1.49)	3 (1.6)	1 (1.25)	
Black or African‐American	30 (11.19)	19 (10.11)	11 (13.75)	
White	215 (80.22)	150 (79.79)	65 (81.25)	
Not available	19 (7.09)	16 (8.51)	3 (3.75)	

### 3.2 Identification of 13 methylation site signature

**FIGURE 2:**
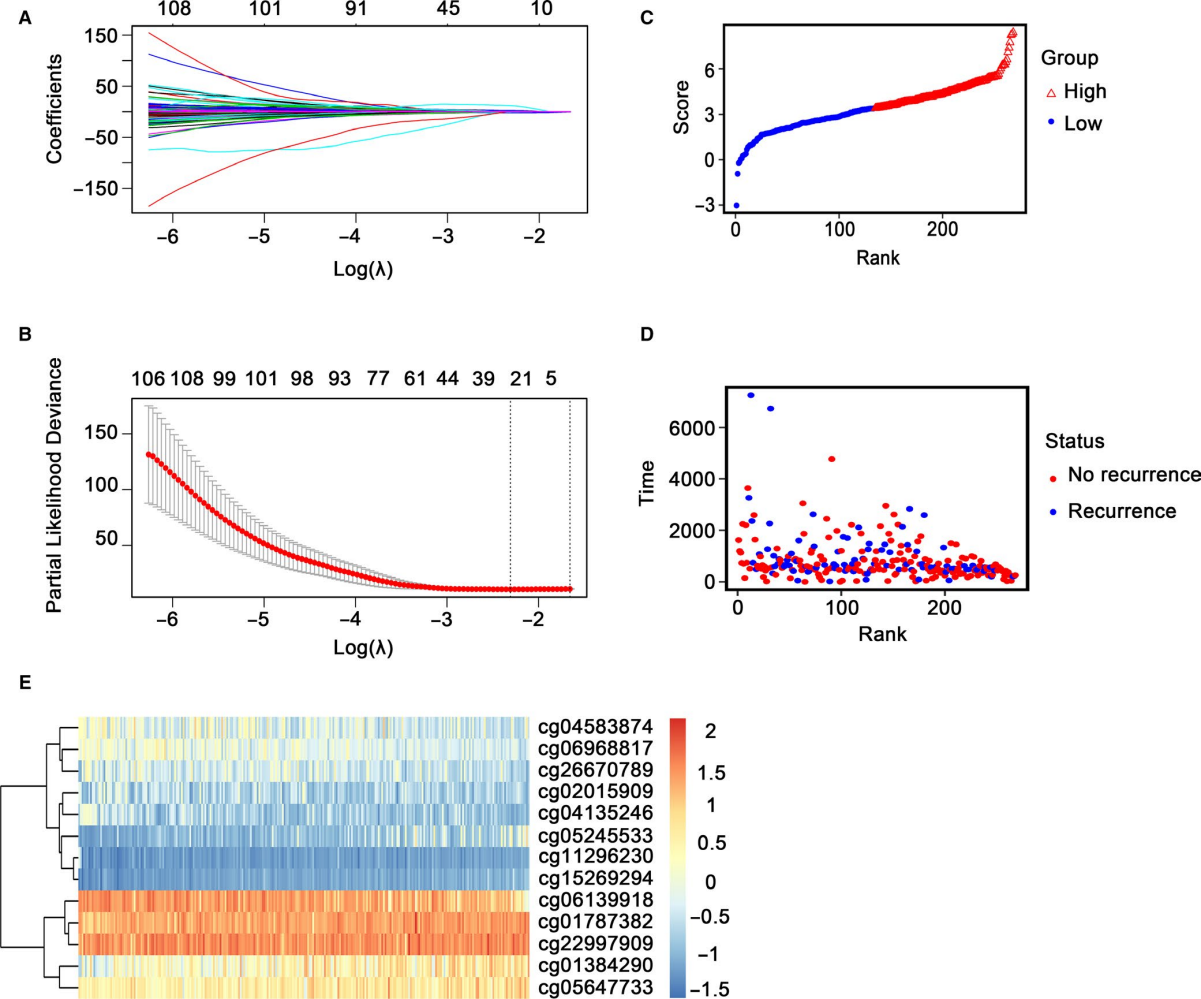
Candidate methylation site selection using the LASSO Cox regression model and construction of the methylation‐related signature. A, 10‐fold cross‐validation for tuning parameter selection in the LASSO model via minimum criteria (the 1‐SE criteria). B, LASSO coefficient profiles of the 530 methylation sites. A coefficient profile plot was produced against log(lambda) sequence. Vertical line was drawn at the value selected using 10‐fold cross‐validation, where optimal lambda resulted in 25 non‐zero coefficients. C, Methylation risk score distribution against the rank of risk score. Median risk score is the cut‐off point. D, Recurrence status of stage I LUAD patients against the rank of risk score. E, Heatmap of 13 methylation site expression profiles of stage I LUAD patients

2372 differentially expressed methylation sites were determined between recurrence and no recurrence groups and were used for univariate Cox proportional hazard regression model, and a total of 530 DNA methylation sites were revealed to be significantly associated with the RFS of stage I LUAD patients (*P* < 0.01) (Table S1). Then, LASSO Cox regression model was acted on these 530 DNA methylation sites and 25 methylation sites were identified as the candidate prognostic indicators for predicting RFS of stage I LUAD patients (Figure 2A,B). Then, multivariate Cox proportional hazard regression model was constructed based on those 25 candidate methylation sites and a risk score formula of 13 methylation sites was created finally: Risk score = 1.56223 × cg01384290 + 4.89164 × cg01787382 − 3.29927 × cg02015909 − 2.98153 × cg04135246 − 4.27669 × cg04583874 + 2.16356 × cg05245533 + 2.85615 × cg05647733 − 2.35513 × cg06139918 − 4.3519 × cg06968817 − 12.46033 × cg11296230 + 13.02145 × cg15269294 + 4.30495 × cg22997909 − 3.12144 × cg26670789. Patients with stage I LUAD were separated into high‐ and low‐risk group with the median risk score as the cut‐off, patients were ranked on the basis of their risk scores (Figure 2C), and the dotplot was drew via their recurrence status (Figure 2D). Result showed that the low‐risk group had a longer RFS than the high‐risk group. Heatmap of 13 methylation sites classified by risk score was shown in Figure 2E, which was corresponding to our previous boxplot (Figure S2).

**FIGURE 3:**
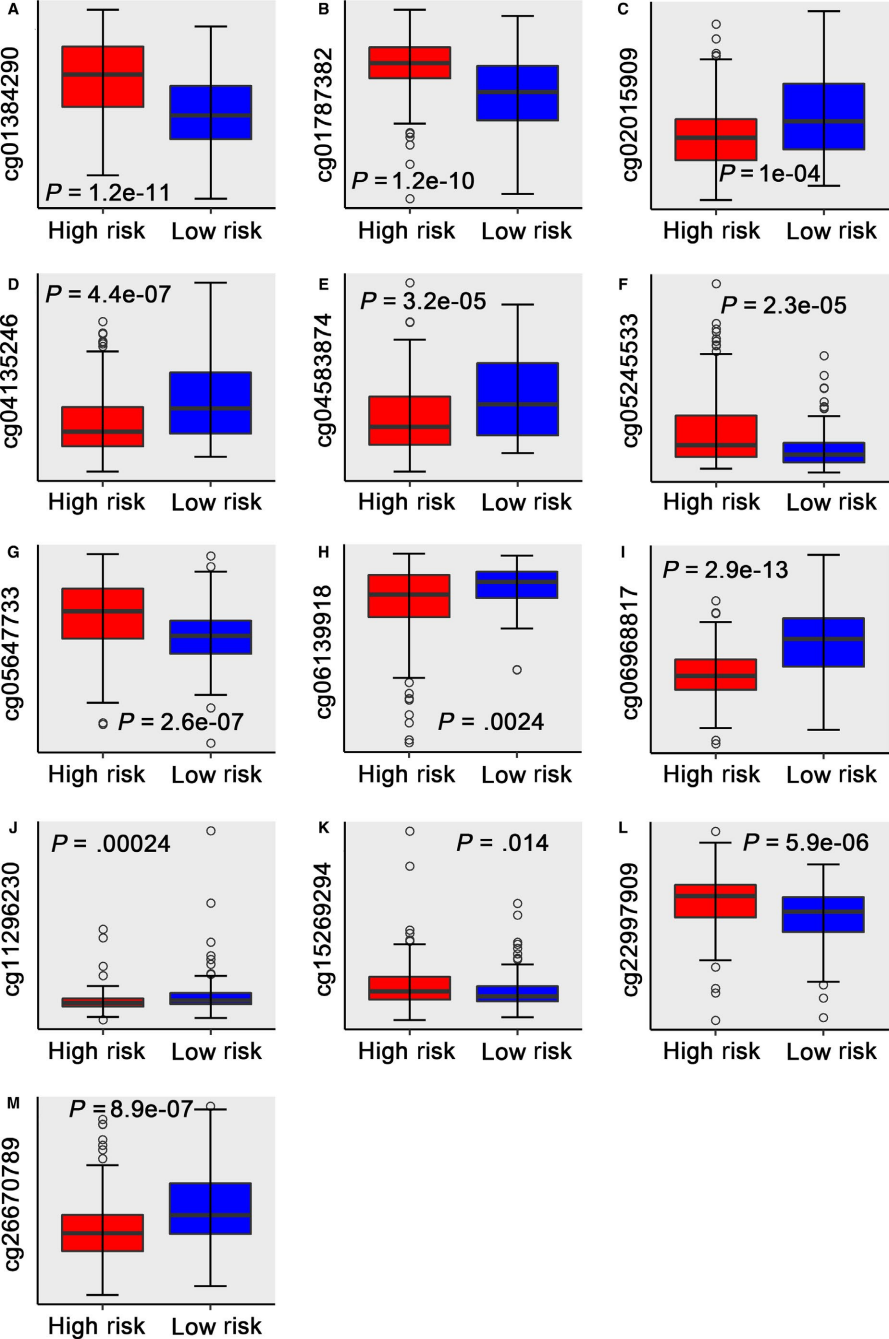
Boxplots of methylation *β* values against risk group in the entire TCGA dataset. ‘High Risk’ and ‘Low Risk’ represent the high‐risk and low‐risk group, respectively. The median risk score was taken as a cut‐off. *Y*‐axis represents the *β*‐value of 13‐DNA methylation sites, respectively. The differences between the 2 groups were estimated by Mann‐Whitney *U* test

Obviously, the hypermethylation levels of cg01384290, cg01787382, cg05245533, cg05647733, cg15269294 and cg22997909 were involved in a higher risk group. Whereas, the hypermethylation levels of cg02015909, cg04135246, cg04583874, cg06139918, cg06968817, cg11296230 and cg26670789 were relevant to a lower risk group (Figure 3) (Figure S1).

### 3.3 Correlation between 13‐DNA methylation signature and patients' RFS in the internal validation and external validation datasets as well as entire TCGA dataset

**FIGURE 4:**
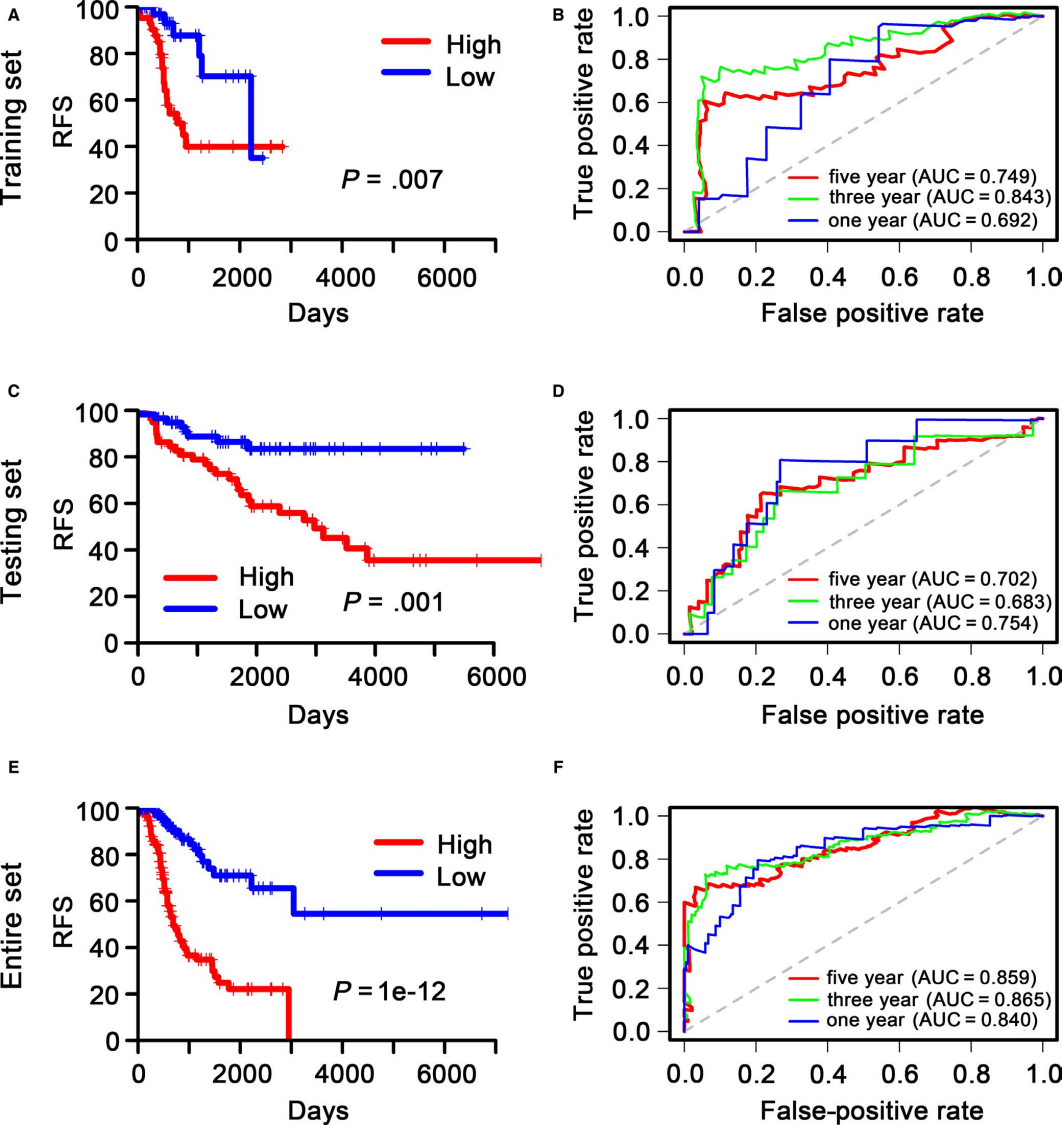
Kaplan‐Meier and ROC analysis of patients with stage I LUAD in internal validation and external validation datasets as well as entire TCGA dataset. A, C and E, Kaplan‐Meier analysis with two‐sided log‐rank test was performed to estimate the differences in RFS between the low‐risk and high‐risk group patients. B, D and F, 1‐, 3‐ and 5‐year ROC curves of the 13‐DNA methylation signature were used to demonstrate the sensitivity and specificity in predicting the RFS of stage I LUAD patients. ‘High’ and ‘Low’ represent the high‐risk score group and low‐risk score group, respectively. The median risk score was taken as a cut‐off

To measure the differences in RFS between the two groups. The Kaplan‐Meier analysis was executed in the internal validation dataset and external validation dataset as well as entire TCGA dataset to evaluate the RFS of patients in the low‐ versus high‐risk cohort, which were classified based on the 13‐DNA methylation signature. The patients with high‐risk scores group had unfavourable RFS in internal validation dataset (*P* = 0.007) (Figure 4A), and similar results were yielded in the external validation dataset (*P* = 0.001) (Figure 4C) and entire TCGA dataset (*P* = 1e−12) (Figure 4E).

### 3.4 Evaluation of the predictive ability of the 13 DNA methylation signature by using ROC analysis

We measured the predictive ability of the 13 DNA methylation signature in predicting RFS via a time‐dependent ROC curve. The AUCs of the 13 DNA methylation signature at 1, 3 and 5 years in internal validation dataset were 0.692, 0.843 and 0.749, respectively (Figure 4B). A high predictive ability was also revealed in external validation dataset (0.754, 0.683 and 0.702) (Figure 4D) and entire TCGA dataset (0.840, 0.865 and 0.859) (Figure 4F), which indicating that the 13‐DNA methylation signature had high ability and had great potential to function as a prognostic signature in clinical practice.

In addition, subgroup analysis was executed by several clinicopathological factors which included age, gender, stage, tumour site and smoking status. The result revealed that the 13‐DNA methylation signature had high predictive value in most of subgroup (Figures [Link], [Link], [Link], [Link], [Link]).

### 3.5 Identification of the 13‐DNA methylation signature‐associated biological pathways

**FIGURE 5:**
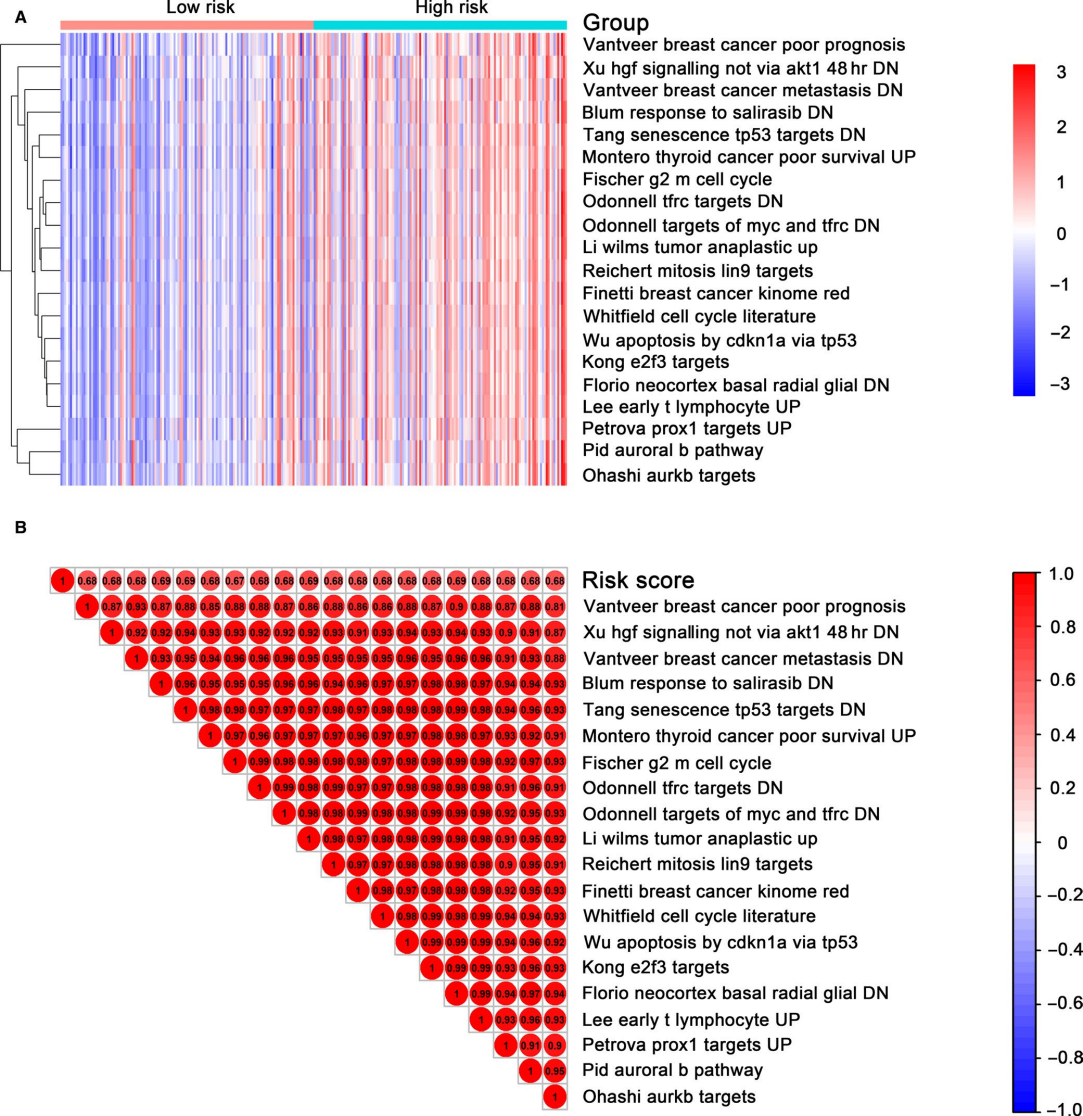
Identification of the 13 DNA methylation signature‐associated biological pathways. A, Heatmap of top 20 enriched pathways associated high‐risk group. B, Correlation graph between risk scores and top 20 pathways

Single‐sample Gene Sets Enrichment Analysis (ssGSEA) was conducted on TCGA LUAD mRNA dataset by using GSVA package^25^ for determination of the 13‐DNA methylation signature‐associated signalling pathways. The patients were divided into low‐ or high‐risk cohorts based on the median methylation score. A few of top 20 pathways including vantveer breast cancer poor prognosis, Xu hgf signature not via AKT1 48HR and vantveer breast cancer metastasis were markedly more activated in the high‐risk patients than that in low‐risk patients (Figure 5A). The trend of the pathways was consistent with the risk score. The relevance of between the risk score and the pathways was further evaluated through correlation analysis. The outcome demonstrated a robust correlation between them (Figure 5B).

### 3.6 Nomogram development and assessment

**FIGURE 6:**
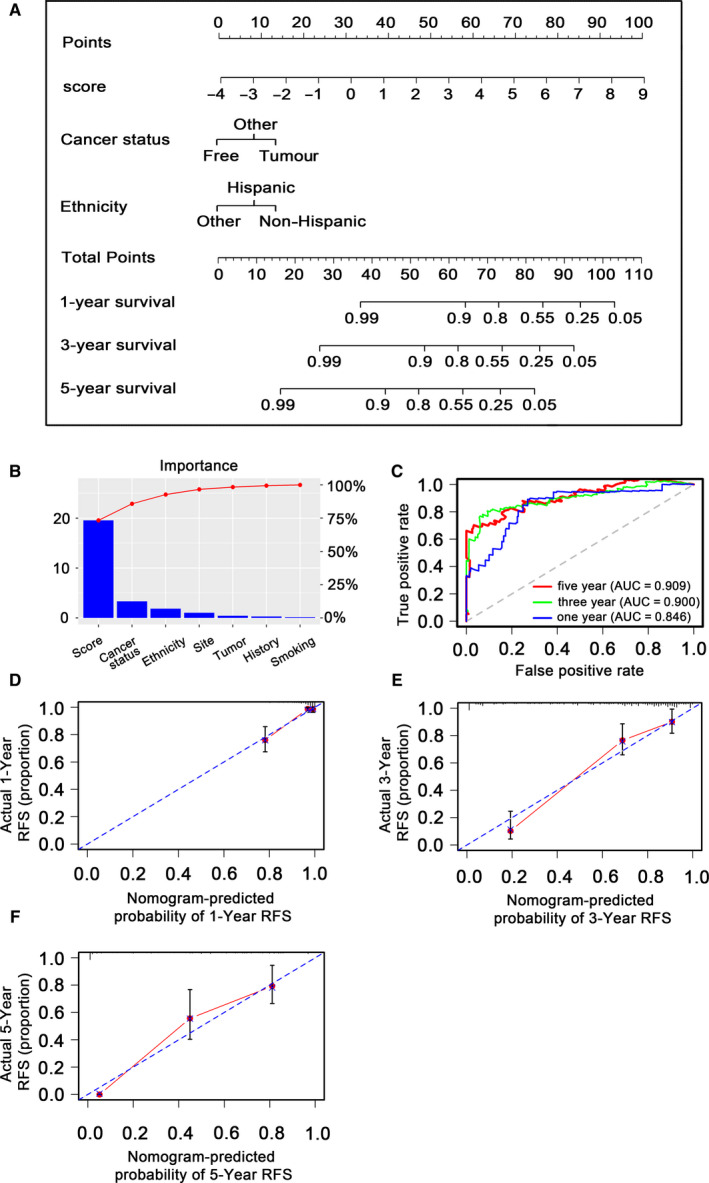
Methylation nomogram for the prediction of stage I LUAD patients' RFS and validation of methylation nomogram in entire TCGA dataset. A, The nomogram was developed in the entire TCGA cohort, with the methylation risk score, cancer status and ethnicity. B, The relative importance of methylation risk score and other clinical indicators. C, 1‐, 3‐ and 5‐year ROC curves for the methylation nomogram. D, E and F, represent the 1‐, 3‐and 5‐year nomogram calibration curves, respectively. The closer the dotted line fit to the ideal line, the better the predictive accuracy of the nomogram is

To assess independence of the 13‐DNA methylation signature for predicting patient RFS, univariate and multivariate Cox model was acted on methylation relevant risk score and some other clinicopathological factors. Hazard ratios (HRs) indicated that the 13‐DNA methylation biomarker was crucially relevant to the RFS of patients (*P* < 0.001, HR 2.52, 95% CI (2.07‐3.07) by the outcome of multivariate Cox regression analysis (Table 2), implying that the signature was an independent prognostic indicator. To predict the prognosis of patients with stage I LUAD with a quantitative method, we built a nomogram (Figure 6A) that integrated the 13‐DNA methylation marker and the conventional clinicopathological factors which produced significant P value in multivariate Cox model to predict stage I LUAD patients' RFS. The importance of variables obtaining significant P value in univariate COX analysis was present in Figure 6B. The result showed that C‐index (0.812, 95%CI: 0.767‐0.857), AUC (0.846, 0.900 and 0.909) (Figure 6C) and calibration plot yielded a high value, respectively (Figure 6D‐F), which strongly demonstrated the reliability of the nomogram served as a significant model for predicting the RFS of stage I LUAD.

**TABLE 2:** Univariate Cox regression analysis and multivariate Cox regression analysis outcome based on methylation risk score and other clinical factors

Univariate Cox analysis					Multivariate Cox analysis			
ID	HR	HR.95L	HR.95H	*P* value	HR	HR.95L	HR.95H	*P* value
Score	2.718281827	2.257482625	3.27313974	4.99E−26	2.519216037	2.070251915	3.065544535	2.80E−20
Sex	1.190788066	0.785542651	1.805091317	0.410678663	1.234569867	0.783432222	1.94549409	0.363812717
T	1.414801719	0.924381171	2.165409646	0.110071377				
N	3.71E−08	0	Inf	0.993929691				
M	0.978813086	0.627736375	1.526237917	0.924724548				
Cancer status	0.495199898	0.374942683	0.654027803	7.36E−07	0.61831797	0.472305909	0.809469254	0.000468889
Age	1.007645831	0.994324503	1.021145631	0.261973795				
Anatomic neoplasm subdivision	1.1414812	1.026606451	1.269210151	0.01447795	1.102553661	0.984284201	1.235034122	0.09172864
Ethnicity	0.607929916	0.367987477	1.004324347	0.052002005	0.535327155	0.325704964	0.879861206	0.013708055
Location in lung parenchyma	0.800719177	0.548018068	1.169945367	0.250676677				
The number of pack‐years smoked	1.007926688	1.001037526	1.014863261	0.024051348	1.001385061	0.993810733	1.009017117	0.720871674
Other family disease history	0.645154686	0.34305619	1.213283952	0.173823123	0.802567865	0.412774092	1.560454469	0.516783907
Race	1.204479117	0.885896961	1.637628311	0.235231162				
Residual tumour	0.96206214	0.616497209	1.501326443	0.864745533				
Tobacco smoking history	1.086624927	0.88332982	1.336707655	0.431810059				

### 3.7 Comparison with other known gene signatures

**FIGURE 7:**
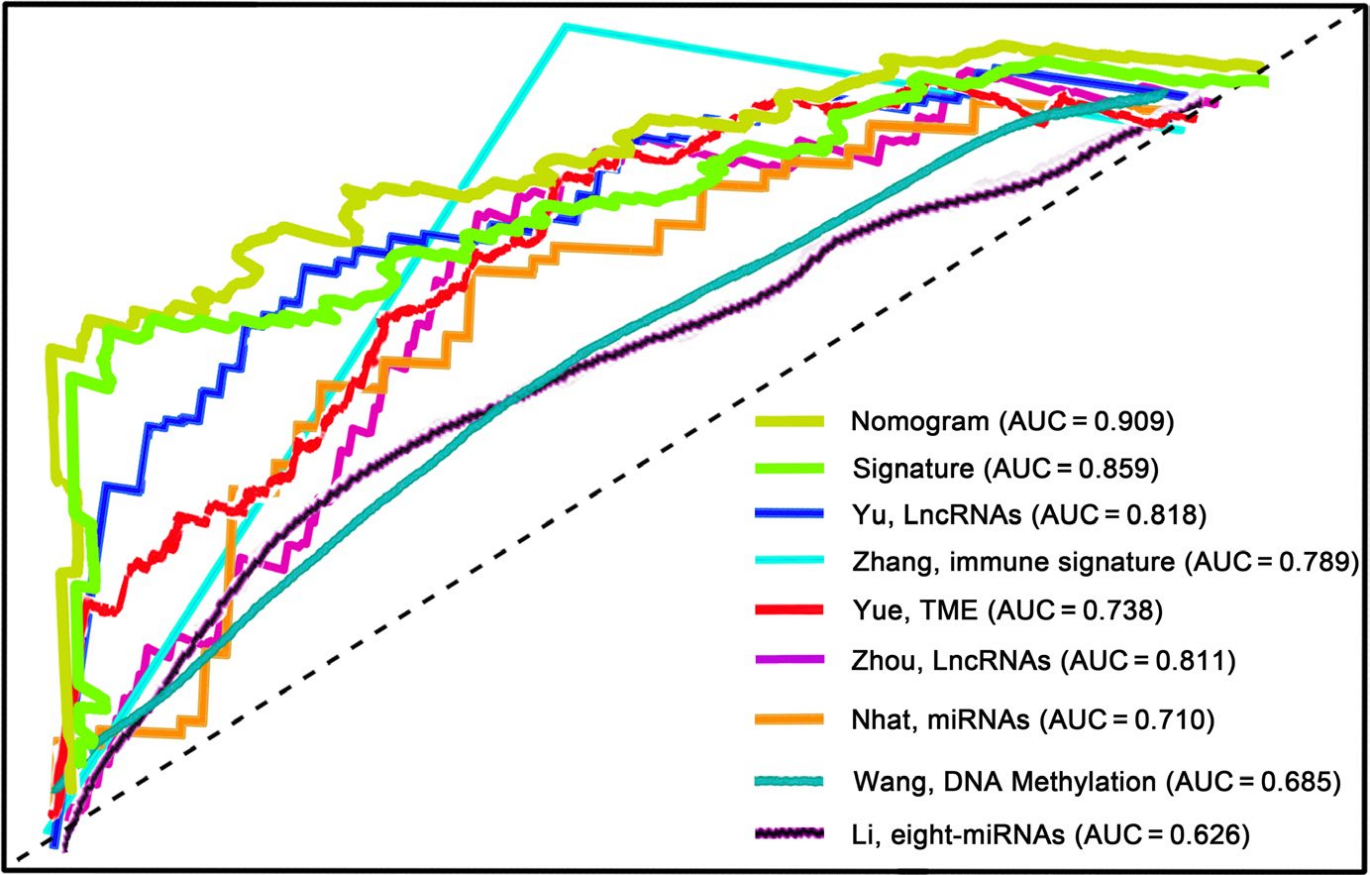
ROC curves show the sensitivity and specificity of the methylation‐associated nomogram and other known biomarkers in predicting the prognosis of stage I LUAD patients

A comparison of our nomogram and signature with other known prognostic hallmarks was performed to assess the robustness of our markers. In order to exclude the impact of heterogeneity, all of these hallmarks that were developed based on TCGA database were included. The markers for predicting all stages or early‐stage LUAD patients' prognosis were also included in our study because the number of biomarkers for stage I LUAD patients' prognosis was limited. The result demonstrated that both our nomogram and signature yielded remarkably better performance in the prediction of stage I LUAD patients' RFS (Figure 7). The AUCs of the nomogram and the signature in our study at 5 years were 0.909 and 0.859 respectively, which was distinctly higher than that of other biomarkers.^26^, ^27^, ^28^, ^29^, ^30^, ^31^, ^32^ The larger the AUC value of a biomarker, the better the predictive ability of the hallmark, which made it clear that our nomogram as well as methylation signature outperformed other signatures in predicting stage I LUAD patients' prognosis.

## 4 DISCUSSION

Early‐stage (IA‑ⅡB) NSCLC accounts for only 25%‐30% of all lung cancers.^4^ Surgery remains the major treatment for operable and resectable stage I LUAD. Whereas, about 20% of patients with stage I LUAD yield cancer recurrence after surgery,^5^ which generates an enormous challenge for public health worldwide. Determination of novel prognostic predictors and construction of more reliable prognostic models are urgently needed.

Multiple molecular markers have been shown to predict the prognosis in various tumours.^33^, ^34^ Numerous researches showed that DNA methylation may serve as underlying prognostic biomarkers. For example, the methylation of PCDH19 served as a hallmark for predicting a poor prognosis of hepatocellular cancer.^35^ The methylation of DFNA5 yielded strong potential as a prognostic hallmark for breast carcinoma.^36^ In the present study, we analysed the whole‐genome methylation profiles of tumour tissues from patients with lung cancer in TCGA database and GEO database to unearth DNA methylation hallmarks for predicting recurrence risk in stage I LUAD. The capacity of methylation factors as molecular prognostic markers was evaluated via Kaplan‐Meier approach and receiver operating characteristic (ROC) analysis. In addition, a nomogram was generated to assess the robustness of the DNA methylation signature for predicting stage I LUAD patients' RFS. The result showed that C‐index, ROC and calibration plot performed well in our study. We successfully developed a DNA methylation‐associated nomogram, enabling prediction of stage I LUAD patients' RFS.

In our study, the selected 13 methylation sites were projected into 6 genes: LY6H, CES8, TMEM200B, NUP155, MECOM and NUAK1. Researchers have reported that the above 6 genes may be significant in cancer development. For instance, Luo et al reported that distinct lymphocyte antigen‐6 (Ly6) family members such as Ly6D, Ly6E, Ly6K and Ly6H may promote tumorigenesis and clinical result.^37^ A recent study revealed that expression of TMEM200B was significantly relevant to overall survival of non‐small cell lung carcinoma.^38^ Holzer et al revealed that nucleoporin nup155 served as part of the p53 network in liver cancer.^39^ Tang et al suggested that t(3;8)(q26.2;q24) often results in MECOM/MYC rearrangement and is commonly related to therapy‐related myeloid neoplasms and/or disease development.^40^ Liu et al reported that expression level of NUAK1 played a significant role in the prognosis of human nasopharyngeal carcinoma.^41^ The result demonstrated that the 6 genes associated with these 13 sites played important roles in cancer progression.

To further explore the predictive ability of our nomogram, a comparison was performed among several significant molecular signatures which were employed for predicting prognosis in stage I LUAD. As there are few studies discovering signatures for predicting RFS of stage I LUAD, the studies for all stages or early‐stage LUAD patients' prognosis also included in our comparison. The AUCs of the nomogram and the signature in our study were remarkably larger than that of other molecular signatures, indicating that our markers outperformed other hallmarks. In particular, the AUC of the nomogram is greater than that of the signature in our study, suggesting that the combination of the risk score with clinical factors is more promising than the methylation signature alone in predicting the RFS of stage I LUAD patients' prognosis.

A nomogram that integrated the 13‐DNA methylation signature and the conventional clinicopathological factors was built to predict stage I LUAD patients' RFS. The research was the first to indicate the transformative application of combining clinical and molecular factors for utility beyond simple classification in the field of personalized prediction for stage I LUAD. According to our established and confirmed models which are publicly available, clinicians may integrate clinical factors and molecular markers to identify a personalized therapy for stage I LUAD patients, which suggests a significant improvement in the field of personalized management for stage I LUAD patients. In addition, the results might facilitate the development of effective biomarkers in clinical practice.

There were also a few limitations in our study. Firstly, in addition to the clinicopathological factors collected in both TCGA and GEO databases, more clinical factors may be used in the nomogram model. In addition, 13‐DNA methylation signature remained to be verified and examined in clinical practice. Finally, a long time was essential for applying it in clinical practice due to a high cost for methylation test. Despite the limitations mentioned above, there were still several superiorities in our study. Firstly, both internal and external validation sets were included to examine the value of the 13‐DNA methylation signature, which indicating the robustness of our model across multiple studies. Besides, LASSO method was used to filter variables between univariate and multivariate Cox analysis, eliminating the interference of the potential multicollinearity in the present study, which made our result more reliable. Moreover, we successfully established a DNA methylation‐associated nomogram combining clinical factors and molecular markers to predict the RFS of patients with stage I LUAD in an effective quantitative approach. We can unearth the exact recurrence probability of the patients through nomogram, while many other studies discovered hallmarks which only determined whether the patient will relapse or not, which demonstrated the potential clinical utility of our model.

In conclusion, the whole‐genome methylation profiles of tumour tissues from patients with stage I LUAD were obtained from TCGA database and GEO database and a predictive risk model for RFS based on methylation of DNAs was established and examined via a bioinformatics approach. Our model displayed strong predictive performance in both TCGA dataset and GEO dataset, which indicated a potential clinical application value of our model and may give us a new direction in understanding clinical diagnosis and treatment. Nevertheless, further larger‐scale, well‐designed and multi‐platform studies should be conducted to confirm these findings before the application of our nomogram for RFS prediction of stage I LUAD.

## CONFLICT OF INTEREST

The authors declare no conflict of interest.

## AUTHOR CONTRIBUTIONS

JC Cheng and P Zhao contributed to literature search; JC Cheng and P Zhao contributed to figures; XX Ma and HY Chen contributed to study design; L Li and KX Tao contributed to data collection; XX Ma and HY Chen contributed to data analysis; L Li and JC Cheng contributed to data interpretation; XX Ma contributed to writing original draft; HY Chen, XX Ma, JC Cheng, P Zhao, L Li and KX Tao contributed to writing draft.

## Supporting information

Fig S1

Fig S2

Fig S3

Fig S4

Fig S5

Fig S6

Fig S7

Table S1

Supplementary Material

## Data Availability

The data that support the findings of this study are available in the GEO database and TCGA data.
