# Supplementary figures and images for: DNA methylation profiling to predict recurrence risk in stage Ι lung adenocarcinoma: Development and validation of a nomogram to clinical management
Source: J Cell Mol Med. 2020 Jun 12;24(13):7576–89. doi: 10.1111/jcmm.15393 (PMC7339160; doi:10.1111/jcmm.15393)

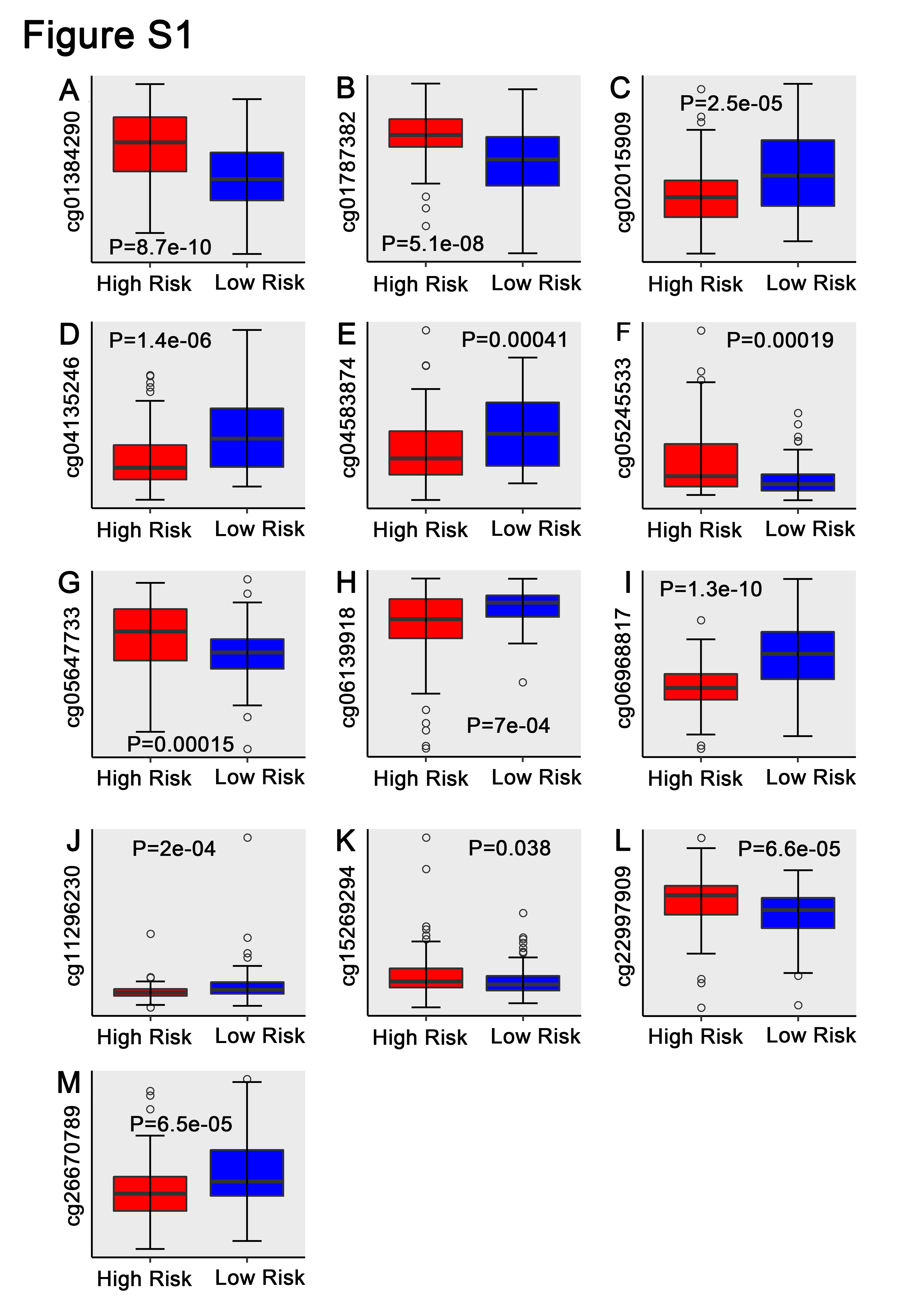

Supplement: Supplementary file 1 — Fig S1 [file JCMM-24-7576-s001.tif]

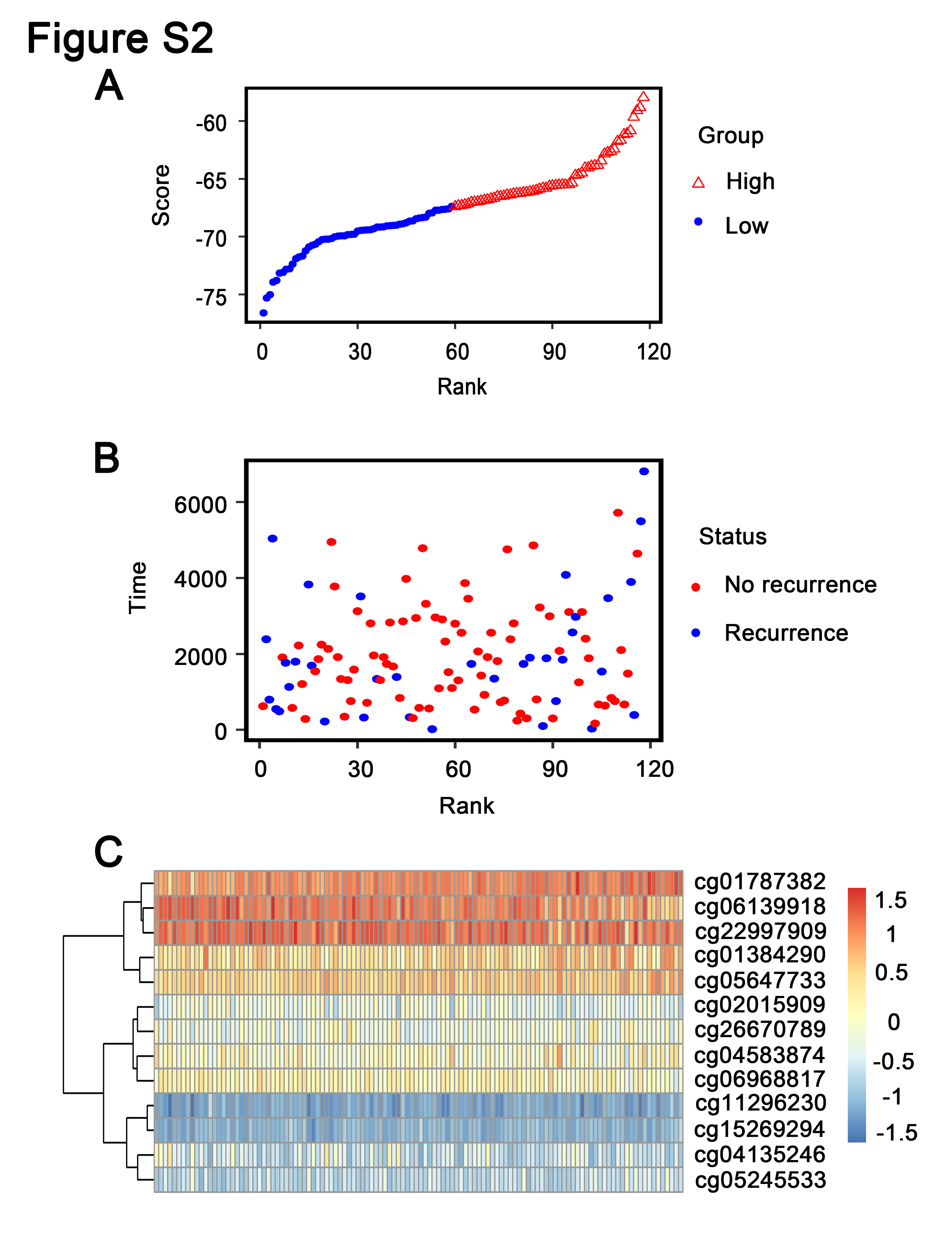

Supplement: Supplementary file 2 — Fig S2 [file JCMM-24-7576-s002.tif]

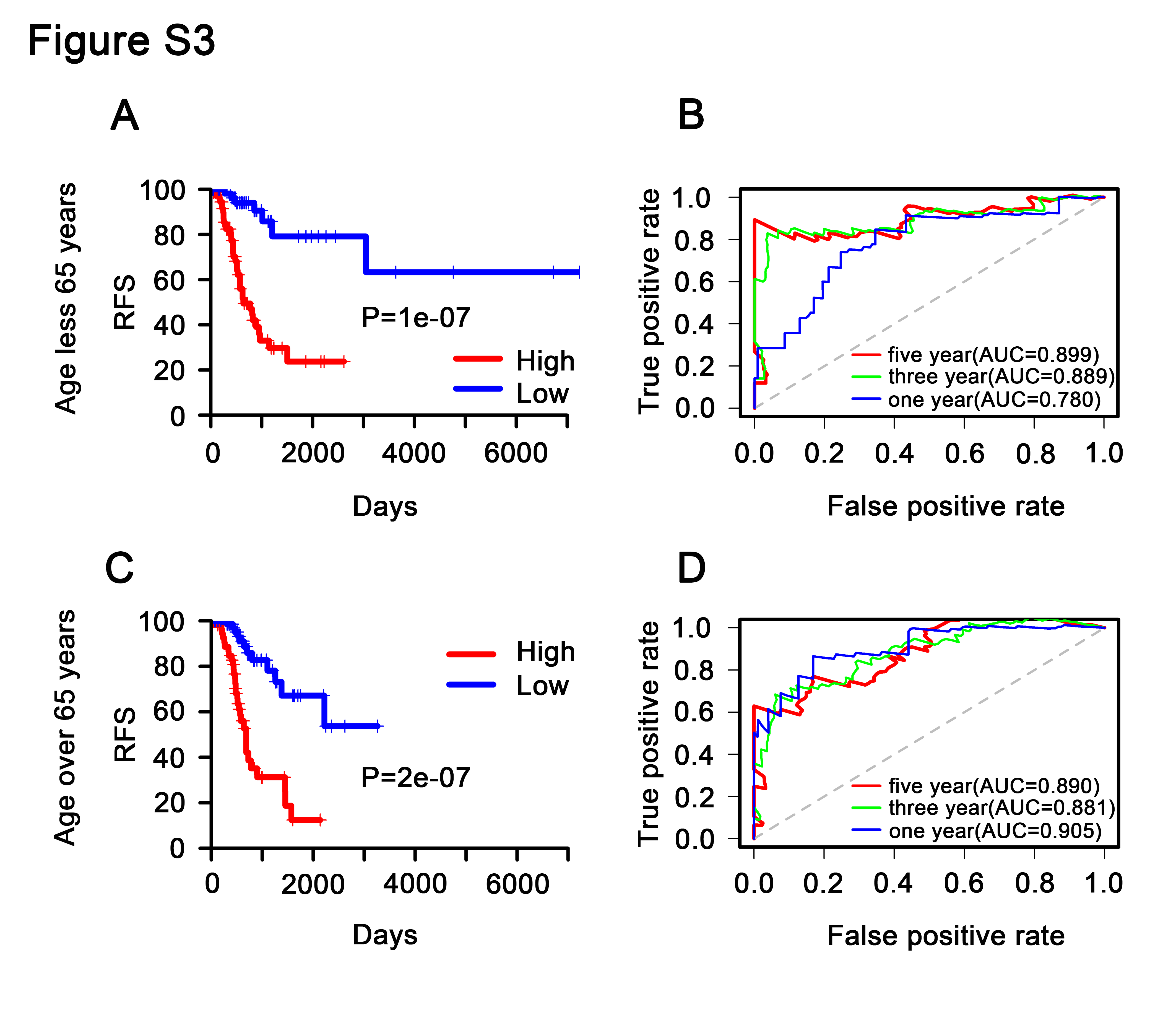

Supplement: Supplementary file 3 — Fig S3 [file JCMM-24-7576-s003.tif]

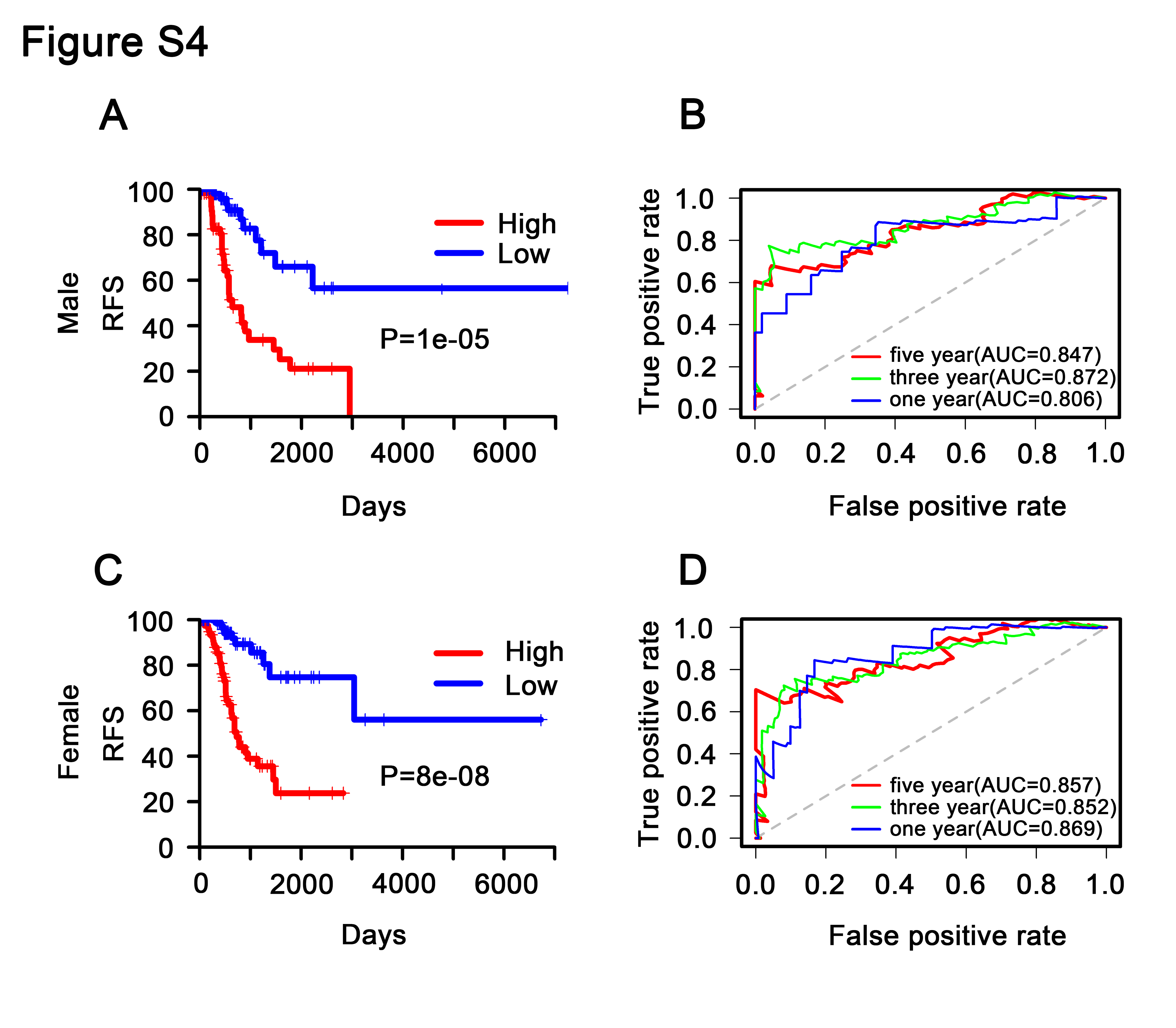

Supplement: Supplementary file 4 — Fig S4 [file JCMM-24-7576-s004.tif]

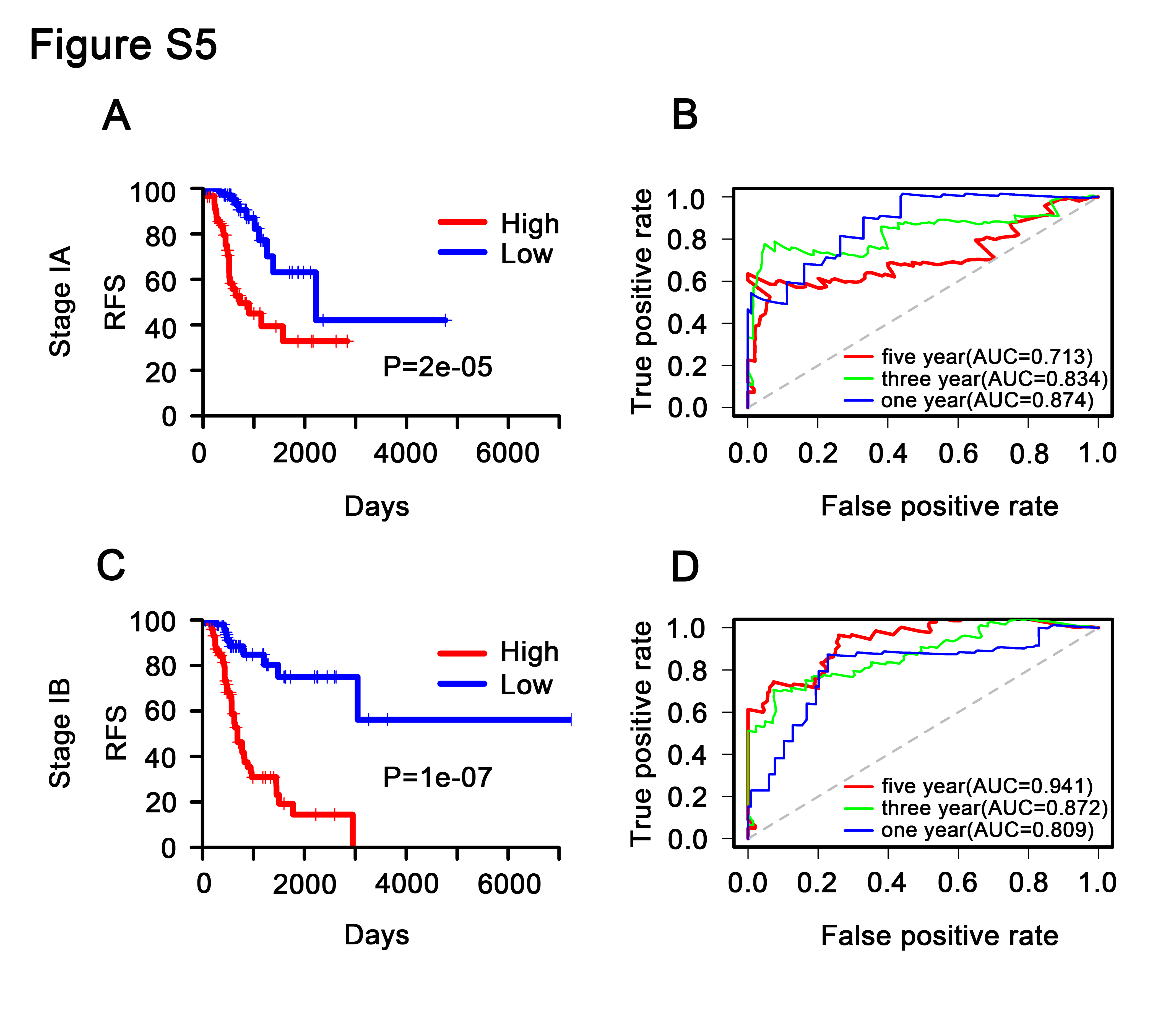

Supplement: Supplementary file 5 — Fig S5 [file JCMM-24-7576-s005.tif]

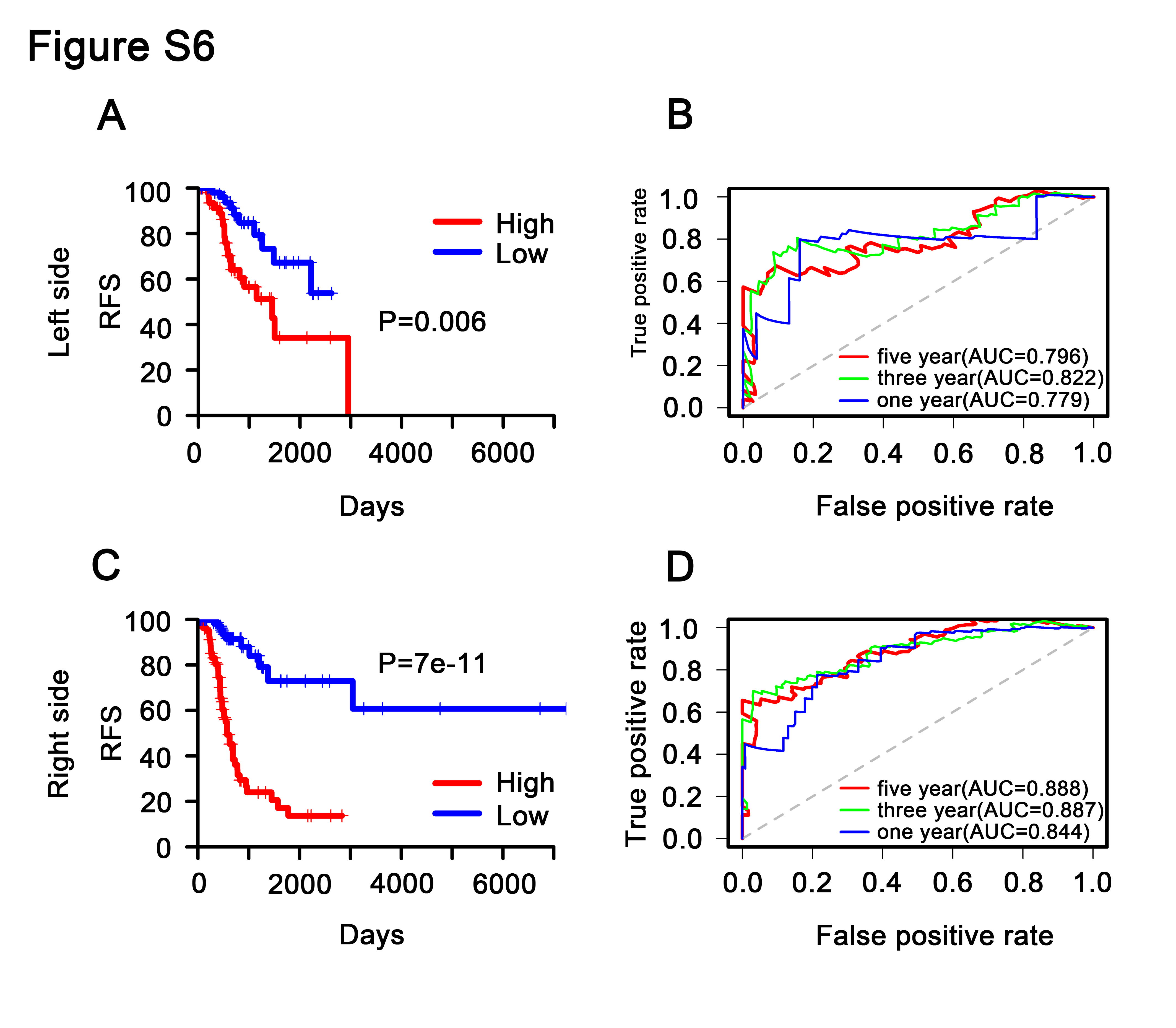

Supplement: Supplementary file 6 — Fig S6 [file JCMM-24-7576-s006.tif]

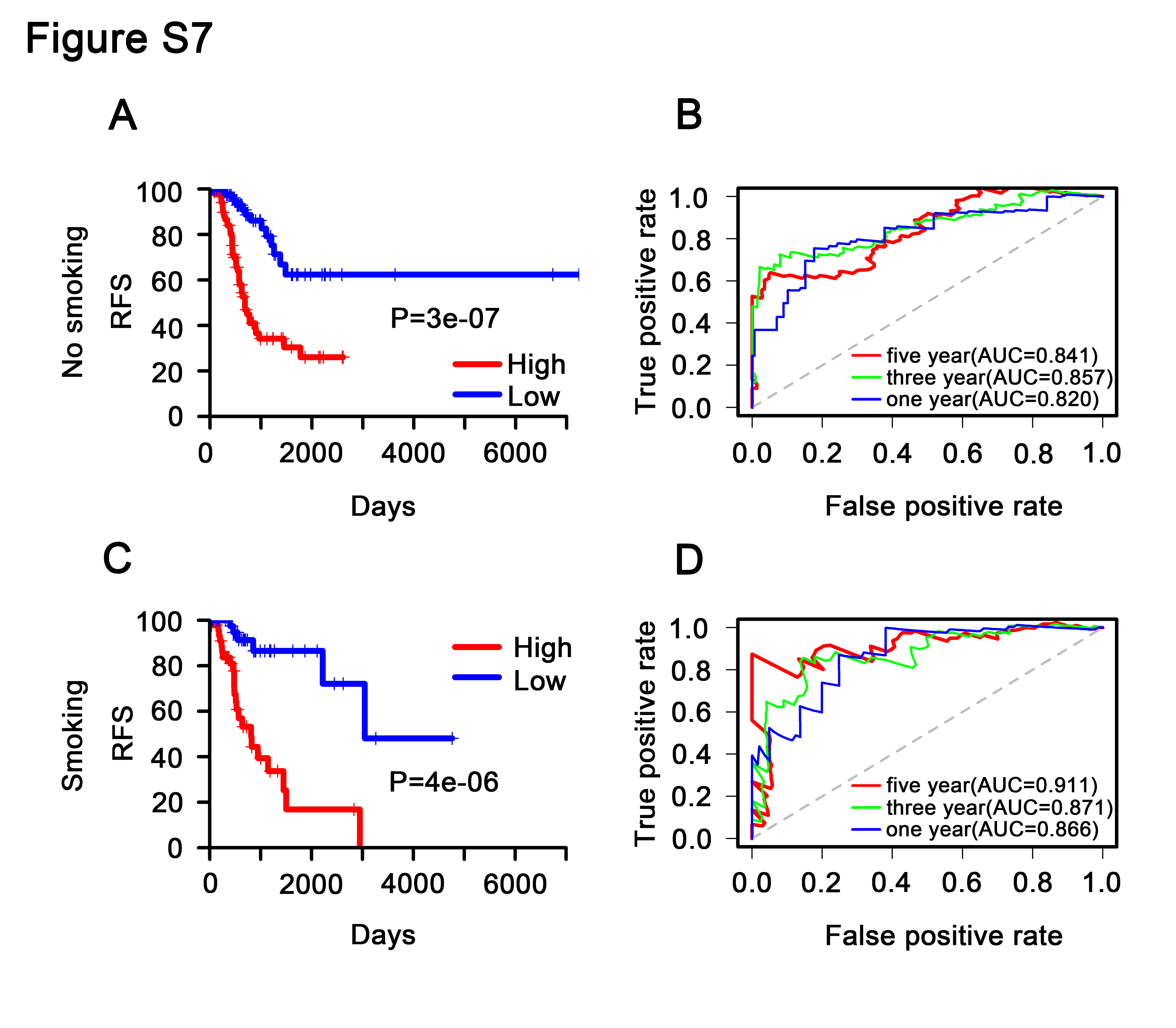

Supplement: Supplementary file 7 — Fig S7 [file JCMM-24-7576-s007.tif]
