# Supplementary material for: DNA methylation profiling to predict recurrence risk in stage Ι lung adenocarcinoma: Development and validation of a nomogram to clinical management
Source: J Cell Mol Med. 2020 Jun 12;24(13):7576–89. doi: 10.1111/jcmm.15393 (PMC7339160; doi:10.1111/jcmm.15393)
Supplement: Supplementary file 9 — Supplementary Material [file JCMM-24-7576-s009.docx]

**Supplementary Figure and Table Legends**

**Figure S1. Boxplots of 13 methylation β values against risk group in GSE39279.** “High Risk” and “Low Risk” represent the high-risk and low-risk group, respectively. The median risk score was taken as a cutoff. Y-axis represent the β-value of 13-DNA methylation sites respectively. The differences between the 2 groups were estimated by Mann-Whitney U test.

**Figure S2**. **Methylation risk score analysis of 118 stage I LUAD patients in GSE39279.** **(A)** methylation risk score distribution against the rank of risk score. Median risk score is the cut-off point. **(B)** Survival status of stage I LUAD patients. **(C)** Heatmap of 13 methylation sites expression profiles of stage I LUAD patients.

**Figure S3. Kaplan-Meier and ROC analysis of patients with stage I LUAD in sub-groups according to age, respectively. (A, B)** Age less 65 years sub-group. **(C, D)** Age over 65 years sub-group. “High” and “Low” represent the high risk score group and low risk score group, respectively. The median risk score was taken as a cutoff. “RFS” represent the relapse-free survival.

**Figure S4. Kaplan-Meier and ROC analysis of patients with stage I LUAD in sub-groups according to sex, respectively.** **(A, B)** Male sub-group. **(C, D)** Female sub-group. “High” and “Low” represent the high risk score group and low risk score group, respectively. The median risk score was taken as a cutoff. “RFS” represent the relapse-free survival.

**Figure S5. Kaplan-Meier and ROC analysis of patients with stage I LUAD in sub-groups according to stage, respectively.** **(A, B)** IA stage sub-group. **(C, D)** IB stage sub-group. “High” and “Low” represent the high risk score group and low risk score group, respectively. The median risk score was taken as a cutoff. “RFS” represent the relapse-free survival.

**Figure S6. Kaplan-Meier and ROC analysis of patients with stage I LUAD in sub-groups according to anatomic site, respectively.** **(A, B)** Left side sub-group. **(C, D)** Right side sub-group. “High” and “Low” represent the high risk score group and low risk score group, respectively. The median risk score was taken as a cutoff. “RFS” represent the relapse-free survival.

**Figure S7. Kaplan-Meier and ROC analysis of patients with stage I LUAD in sub-groups according to smoking status, respectively.** **(A, B)** No smoking sub-group. **(C, D)** Smoking sub-group. “High” and “Low” represent the high risk score group and low risk score group, respectively. The median risk score was taken as a cutoff. “RFS” represent the relapse-free survival.

**Table S1.** Significantly enriched GO terms based on genes located at 2372 differentially expressed methylation sites.
